# Total Protein Intake in Patients with PKU: Adequacy Evaluation According to the European PKU Guidelines from 2017

**DOI:** 10.3390/nu15234883

**Published:** 2023-11-22

**Authors:** Melanie Gomes, Manuela Ferreira Almeida, Catarina Sousa Barbosa, Maria Inês Gama, Maria Peres, Élia Pinto, Anita MacDonald, Júlio César Rocha

**Affiliations:** 1 Nephrocare Portugal, Fresenius Medical Care Nutrition Departament, Rua Professor Salazar de Sousa, Lote 12, 1750-233 Lisboa, Portugal; melanievgomes@gmail.com; 2 Centro de Genética Médica Jacinto Magalhães, Centro Hospitalar Universitário de Santo António, 4099-028 Porto, Portugal; manuela.almeida@chporto.min-saude.pt (M.F.A.); catarina-s-@hotmail.com (C.S.B.); 3 Centro de Referência Para as Doenças Hereditárias do Metabolismo, Centro Hospitalar Universitário de Santo António, 4099-028 Porto, Portugal; 4 Unidade Multidisciplinar de Investigação em Biomedicina, Instituto de Ciências Biomédicas Abel Salazar, Universidade do Porto, 4050-313 Porto, Portugal; 5 University Hospital Southampton NHS Foundation Trust, Southampton SO16 6YD, UK; inesrgama@gmail.com; 6 National Institute of Health Doutor Ricardo Jorge, Av. Padre Cruz, 1649-016 Lisboa, Portugal; mariacpperes@gmail.com; 7 Faculdade de Medicina, Universidade do Porto, FMUP, Alameda Prof. Hernâni Monteiro, 4200-319 Porto, Portugal; eliajoana17@gmail.com; 8 Birmingham Children’s Hospital, Birmingham B4 6NH, UK; anita.macdonald@nhs.net; 9 CINTESIS@RISE, NOVA Medical School, Faculdade de Ciências Médicas, NMS, FCM, Universidade Nova de Lisboa, Campo Mártires da Pátria 130, 1169-056 Lisboa, Portugal; 10 NOVA Medical School, Faculdade de Ciências Médicas, NMS, FCM, Universidade Nova de Lisboa, Campo Mártires da Pátria 130, 1169-056 Lisboa, Portugal

**Keywords:** protein intake adequacy, protein intake, natural protein, protein substitutes, phenylketonuria, phenylalanine, European PKU guidelines

## Abstract

In PKU, the protein requirements are contentious. In 2018, we evaluated the protein intake in patients with PKU. Ninety-nine early treated patients aged 19.3 ± 8.2 years (54% males) were studied. A total of 24 had hyperphenylalaninemia (HPA), 48 mild and 27 classical PKU. All had an annual nutritional status evaluation. A total of 83% were on diet therapy only, and 17% were on diet with tetrahydrobiopterin therapy. Anthropometry, metabolic control and nutritional intake [total protein (TP, g/kg), natural protein (NP, g/kg), protein equivalent from protein substitutes (PE, g/kg)] were collected. TP adequacy (TPA) was calculated as a % of WHO (2007) safe levels of protein intake. Results were compared with the European PKU Guidelines (EPG). The median % contribution NP of TP intake was 53% [31–100]. Most patients (78%) had a TP intake above the EPG recommendations. The median TPA was 171% [146–203], with 79% [51–165] from NP and 84% [0–109] from PE. A TPA of 100–140% was observed in 16 (16%) patients. Only n = 6 (6%) patients had a TPA < 100%. These results emphasize the heterogeneity of PKU. More research is needed to understand the necessity of a single protein recommendation for all, as a ‘one-size-fits-all’ solution might not be appropriate.

## 1. Introduction

Phenylketonuria (PKU) is an inborn error of protein metabolism. This autosomal recessive disorder is defined (98–99% of patients) by the partial or complete enzymatic inactivity of the hepatic enzyme, phenylalanine hydroxylase (PAH) [1]. An oral pharmacological treatment, tetrahydrobiopterin (BH4), may help a sub-group of patients by lowering blood phenylalanine (Phe) levels, and improving dietary Phe tolerance [2,3]. An enzymatic therapy, pegvaliase, enables greater dietary relaxation in patients but is only available in a few countries and suitable for adults only [4]. Therefore, a lifelong dietary restriction remains the main management strategy [5]. The dietary goal is to optimize Phe tolerance, whilst maintaining metabolic control within the target therapeutic range [6]. A Phe-restricted diet is based on natural protein (NP) restriction supplemented with protein substitutes (PS), free or low in Phe, in order to achieve protein recommendations and attain satisfactory growth by improving protein anabolism. PS are also essential to maintain acceptable metabolic control in patients with PKU and usually have added vitamins, minerals and essential fatty acids such as docosahexaenoic acid (DHA) [6,7,8].

The protein recommendations in PKU are contentious. Throughout the last 70 years in PKU, protein requirements have been debated [6]. In 2014, the American College of Medical Genetics and Genomics (ACMG) guidelines suggested a protein intake of 120–140% of the recommended daily allowance (RDA) age specific from 4 years to adulthood [9]. In 2017, the European PKU guidelines (EPG) recommended 140% (20% to compensate for the digestibility losses from amino acids and the other 20% to help optimize blood Phe control) of the 2007 World Health Organization (WHO/UNU/FAO) protein requirements [6,10]. Both the 2014 ACMG and 2017 EPG suggested that daily total protein (TP) requirements should be higher than the general population [6,9]. PS based on amino acids has a high amino acid oxidation rate and decreased protein retention compared to NP sources, leading to less efficient protein utilization [11,12,13]. In addition, an adequate energy intake is essential in order to optimize the nitrogen retention of protein sources [6]. Although the optimal protein:energy ratio to optimize growth and protein retention in PKU is unclear, a value of 1.5 to 2.9 g of protein/100 kcal has been suggested [14].

Furthermore, the daily dose of protein equivalent (PE) from PS is dependent on Phe tolerance, age, weight, adherence to PS and metabolism efficacy of either NP or PS [6,11,15]. The type of protein source prescribed (amino acids vs. peptide) will also influence the efficiency of protein absorption and retention, and therefore may alter protein requirements [11,12]. Innovative protein substitutes such as prolonged-release PS (an amino acid mixture engineered with a modified-release technology) has an improved kinetic profile compared with amino acids and evidence suggests in non-PKU human studies and it is associated with better nitrogen retention [16]. Glycomacropeptide (GMP), a protein substitute derived from cheese production, is a peptide that contains a small amount of Phe. Many studies show that GMP is associated with an increase in blood Phe levels, but the majority show no statistically significant change except in studies in children [17]. Van Calcar found higher insulin rates and lower blood urea nitrogen postprandial in a group of patients with PKU, leading to higher protein retention in GMP when compared to amino acids [18]. Development of new PS’s using cutting-edge science and technology is essential in order to provide better treatment options to this population.

The aim of this study was to evaluate protein intake and their sources in patients with PKU according to the disorder severity (PKU classification) compared with EPG recommendations.

## 2. Materials and Methods

### 2.1. Study Design

**Figure 1:**
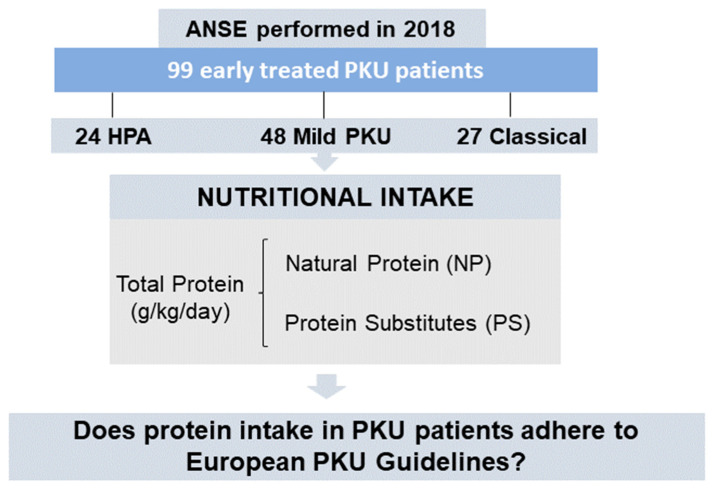
Study design. Abbreviations: ANSE—Annual Nutritional Status Evaluation, PKU—phenylketonuria, HPA—hyperphenylalaninemia, NP—natural protein and PS—protein substitutes.

We performed a cross-sectional, retrospective, descriptive, observational study (Figure 1) with data collected in 2018 from the annual nutritional status evaluation (ANSE) of patients with PKU. Protein intake was calculated and its adequacy was compared with the WHO/UNU/FAO (2007) protein intake safe levels. Protein intake was also compared with the EPG (WHO + 20% and WHO + 40%) recommendations [6,19]. Age, sex, weight, height, body mass index (BMI), disease severity (phenotype), genotype, TP, NP and PS intake from Phe-free amino acid mixtures (AAM), GMP and large neutral amino acids (LNAA) were collected from electronic patient clinical records. Blood [Phe] spots were collected after an overnight fast and analyzed by tandem mass spectrometry (MS/MS) in the hospital laboratory. All patient names were coded to protect their identity and data. There was no control group, as all participants had a diagnosis of PKU or hyperphenylalaninemia (HPA).

### 2.2. Participants

In 2018, 105 patients with PKU were followed-up at Centro Hospitalar Universitário do Porto (CHUPorto), now Centro Hospitalar Universitário de Santo António. The disorder severity was classified according to neonatal screening blood Phe concentrations, described in the Portuguese Consensus: HPA (blood Phe < 360 µmol/L), mild PKU (blood Phe between 360 and 1200 µmol/L) and classical PKU (blood Phe > 1200 µmol/L) [20]. Patients who were late diagnosed, pre-conception or pregnant (n = 6) were excluded.

### 2.3. Eligibility

Only patients diagnosed through newborn screening and with an ANSE protocol scheduled in 2018 were included. ANSE collection included anthropometric data, body composition analysis and nutritional intake.

### 2.4. Data Collection

#### 2.4.1. Anthropometry

Measurements were collected by two experienced nutritionists (J.C.R and M.F.A) from CHUPorto. Patients were weighted on SECA^®^ (Hamburg, Germany) scales (accuracy = 0.5 kg) with light-weight clothes, without shoes or jewelry. Height was measured by a stadiometer (Seca^®^, measured to the nearest millimeter). In pediatric ages (<12 years) and adolescents (12 to 18 years), z-scores were calculated using WHO Anthro^®^ and Antroplus^®^ softwares, and overweight/obesity classified according to recommended cut-offs [10,21]. For adult patients (≥19 years), BMI was defined by categories as referenced in the WHO criteria [22].

#### 2.4.2. Nutritional Intake

Protein intake data were collected from patient clinical records and from self-reported 24 h recalls/food intake history on the day of the ANSE. Dietary intake and type, quantity and timing of PS, meal and snack intake was documented and analyzed by two inherited metabolic disorders nutritionists (J.C.R and M.F.A). TP intake (g/kg/day) was measured and described as the sum of NP intake [natural foods (g/kg/day)] and PE (g/kg/day) from PS. Median TP adequacy (TPA) was calculated as a percentage of WHO/UNU/FAO safe levels of intake and compared to EPG recommendation (WHO + 20%/WHO + 40%). The WHO/UNU/FAO safe levels of protein intake define the protein requirements as 97.5% of a healthy population [10]. Individual patient dietary data was transferred to an Excel database in order to calculate patients nutritional intake. The database contained nutritional information for NP (from Portuguese Food Composition Tables) and PS available in Portugal.

#### 2.4.3. Metabolic Control

Fasting dried blood spots for Phe levels were taken by caregivers/patients at home. Patients and their caregivers had been instructed by a nurse or laboratory assistants about the technique for taking dried blood spots. Blood Phe was collected after an overnight fast and analyzed by tandem mass spectrometry (MS/MS) at the hospital laboratory. Blood Phe during the previous 12 months to the ANSE was collected from the patient electronic clinical record database. These were used to calculate blood Phe median and to analyze the percentage of blood Phe within target range.

Metabolic control was considered satisfactory according to blood Phe targets: 360 µmol/L or 480 µmol/dL in patients below and ≥12 years, respectively, as stated at the Portuguese Nutritional Consensus [20].

#### 2.4.4. Ethical Statement

This study and its data collection were approved by the ethics committee of Centro Hospitalar Universitário do Porto, for the investigation project “Trends in Nutritional Status of patients with phenylketonuria”, with the reference 2015.101 (092-DEFI/087-CES). Participants and parents/caregivers of children gave written informed consent.

#### 2.4.5. Statistical Analysis

SPSS 25 for Windows was used for the statistical analysis. Descriptive statistics were also used with the mean ± SD or median [P25–P75] estimated. The Kolmogorov–Smirnov test was used to verify the normality of variables. Comparison of continuous variables was performed by the Mann–Whitney test. Statistical significance was considered when *p* < 0.05.

## 3. Results

### 3.1. Subjects

The study sample comprised 99 patients, 53 males and 46 females, aged 19.3 ± 8.2 years (range 3–36 years) as described in Table 1. There were 18 (18%) patients under 12 years (pediatric group), with a mean age of 7.2 ± 2.6 years. Phenotypically, there were 24 (24%) patients with HPA, 48 (49%) with mild PKU and 27 (27%) with classical PKU. All subjects were treated with a Phe-restricted diet and had regular nutritional review. Data on anthropometry are described in Table 2. Of the 99 patients, 64 (65%) were prescribed a PS. AAM was the main source of PS in the majority of patients (n = 53, 83%), eight (13%) were exclusively taking GMP, two (3%) patients were taking GMP together with AAM and one (1%) was prescribed LNAA. A sub-group of patients (n = 17, 17%) were taking BH4 with a Phe-restricted diet (1 HPA, 12 mild PKU and 4 classical PKU).

### 3.2. Protein Intake

Total median NP intake was 0.69 [0.43–1.40] g/kg and PS intake was 0.70 [0–0.91] g/kg. Median NP intake provided 53% [31–100] of TP intake whilst PE from PS intake contributed 47% [0–69] of TP intake. Protein intake (g/kg) is described in Table 3—TP, NP and PS sources.

#### 3.2.1. NP Intake Only

A total of 35% (n = 35) of patients were not prescribed a PS and were on a controlled natural protein diet only. Twenty-three were HPA (96% of HPA patients), ten were mild PKU (21% of mild PKU patients) and two classical PKU (7% of classical PKU patients). Both classical patients with PKU were adults (>19 years) and had a median TP intake of 0.39 [0.33–0.44] g/kg from NP sources only. Most BH4-treated patients were prescribed PS (n = 14/17, 82%).

#### 3.2.2. With PS Prescription

A total of 65% (n = 64) of patients were prescribed PS, with NP providing 34% [25–51] of TP intake. These patients had a median PE from PS intake of 0.86 [0.71–1.04] g/kg. Thirty-eight had mild PKU (79% of mild patients) and 28 classical PKU (93% of all classical PKU patients). Only one HPA (4% of HPA) patient took PS (0.22 g/kg). Children taking PS (n = 13) had a median PE intake of 1g [0.83–1.16] g/kg compared to 0.81 [0.71–0.99] g/kg in adults.

The majority were taking PS in three daily doses (n = 57), at breakfast (97%), afternoon (84%) and bedtime (78%).

### 3.3. Protein Adequacy

**Figure 2:**
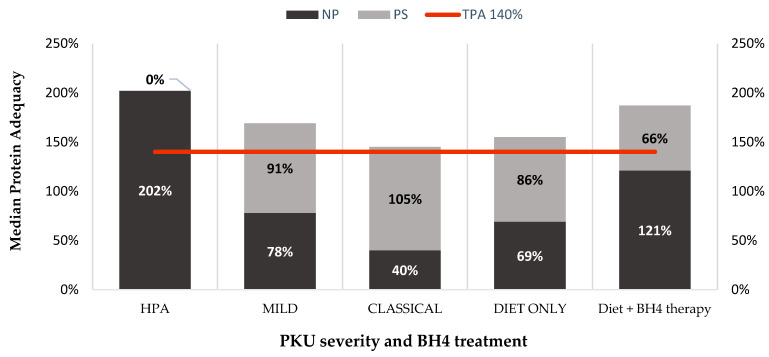
Median protein adequacy from NP and PS according to the different PKU severity, diet only or diet + BH4 therapy. Red line represents the 140% TPA (EPG recommendation) [8]. Abbreviations: PKU—phenylketonuria; HPA—hyperphenylalaninemia; NP—natural protein; PS—protein substitutes; BH4—tetrahydrobiopterin.

The majority of patients (78%) had a TPA above the EPG (WHO + 40%). A total of 17% (n = 17) of patients were children (<12 years) and 61% (n = 60) were patients ≥12 years. The % of TPA according to disease severity is shown on Table 4. The median TPA was 171 [146–203]%, with median NP 79 [51–165]% and PS 84 [0–109]% of the TPA. Table 5 describes the median TPA, PS and NP adequacy according to age, disease severity and type of treatment. Mild forms of PKU were less likely to attain a TPA > 140% compared to HPA group as described on Table 5. Only 16% of HPA and mild form of PKU did not achieve EPG recommendations in contrast of the 37% with classical PKU patients. Figure 2 represents NP and PS contributions to median protein adequacy according to PKU severity, diet only or diet + BH4 therapy.

Patients not taking PS had a median TPA of 199 [150–234]%. In this group, only classical patients had a TPA < 140% (TPA of 46 [40–53]%), HPA and mild PKU patients had a TPA of 200 [165–225]% and 204 [149–244]%, respectively. Median blood Phe in the sub-group of patients who achieved TPA of 140% was within target range (<12 years: 234 [192–270] µmol/L and ≥12 years: 258 [240–288] µmol/L). However, classical PKU patients without PS prescription had a median blood Phe of 834 [726–948] µmol/L.

In the BH4-treated group, most patients had a TPA over 140% (n = 14, 82%), with only three patients with a TPA lower than 140%. TPA was similar when comparing patients on BH4 therapy and patients on diet treatment only: 182% [155–206] vs. 168% [144–202]; *p* = 0.55.

### 3.4. Metabolic Control

The median blood Phe control was within target range according to the Portuguese Consensus (<12 years: median 294 [234–354] µmol/L and ≥12 years: 450 [270–600] µmol/L), as described in Table 6. In patients ≥12 years (n = 81), 43% (n = 35) had a blood Phe > 480 µmol/L with a median of 642 [558–804] µmol/L. Patients with age <12 years had a median of 65% of blood Phe levels within target range and 61% for patients ≥12 years of age (Table 6). Blood Phe control deteriorated with severity of PKU, with only 30% of patients with classical PKU achieving a median blood Phe level within target range. The BH4 group achieved metabolic control according to recommended targets.

#### 3.4.1. Metabolic Control and Patients with TPA < 140%

A TPA of <140% was described in 22 patients (22%). Eight of those patients (36%) had blood Phe metabolic control within target range compared with 14 patients (67%) who were above the recommended blood Phe targets.

In the well metabolically controlled group, only one patient (HPA) was <12 years and four were adolescents (12–18 years) (2 HPA, 1 mild PKU and 1 classical PKU). These patients had a median NP intake of 0.68 [0.32–1.06] g/kg and 0 [0–0.7] g/kg from PS sources. Classical PKU patients were prescribed PS.

In the group with blood Phe levels above target range (n = 14), 6 had mild PKU and 8 classical PKU. This group had a median NP intake of 0.27 [0.23–0.48] g/kg and 0.73 [0.3–0.81] g/kg from PS sources.

#### 3.4.2. Metabolic Control and Patients Taking PS

In patients taking a PS, median blood Phe was within the target range only in patients aged <12 years (n = 12; 322 [236–355] µmol/L compared with 514 [435–676] µmol/L in patients aged ≥12 years). The overall median blood Phe was 394 [249–576] µmol/L.

### 3.5. Natural Protein: Sources

**Figure 3:**
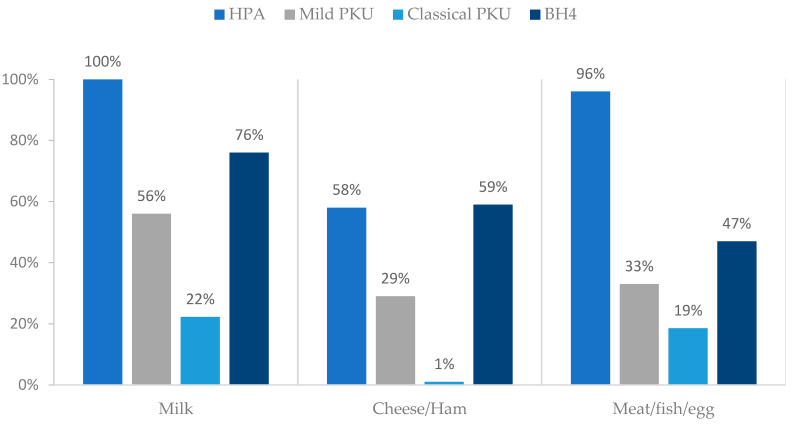
Percentage of consumption of high biological protein sources according to different PKU severity and BH4 treatment. Abbreviations: PKU—phenylketonuria; HPA—hyperphenylalaninemia; BH4—tetrahydrobiopterin.

Milk/yoghurt were ingested by 69% (n = 68) of patients. Pulses (consumed by 30%, n = 30) and high biological protein sources were the least ingested food groups (44%, n = 44 meat/fish/eggs, 33% (n = 33) cheese/ham). More than 58% of HPA patients had high biological NP food sources as described on Figure 3.

NP intake for patients on BH4 therapy (n = 17) was 1.00 [0.79–1.2] g/kg, while for non-BH4 -treated patients was 0.6 [0.35–1.45] g/kg of NP intake (as stated at Table 3).

## 4. Discussion

This study described the TPA in patients with PKU compared with the 2017 EPG. Our main finding was that 78% of patients (77 out of 99) achieved TPA as recommended by the EPG (WHO + 40%) and had a median total protein prescription of 71% more than the WHO/UNU/FAO (2007) safe protein intake. In patients with classical PKU, PS intake was essential to attain TPA. Although 65% of patients were prescribed PS, the majority of protein came from NP in our patient cohort who had mainly HPA or a mild phenotype of PKU (73% of patients). This study described actual protein intake rather than prescribed amount, and low TPA intakes were associated with low adherence with PS in patients with classical PKU.

NP prescription depended on individual Phe tolerance, and when individual tolerance is high, it is important it is obtained from diverse and good quality protein sources [12,23]. Food patterns in PKU patients are influenced by disease severity and nutritional treatment. This is the first paper in our cohort that describes protein quality intake. Patients with milder PKU are able to eat more and varied animal protein sources and have a different NP/PS ratio in comparison to classical PKU, as observed in our study. In addition, patients prescribed sapropterin had a higher NP intake, commonly tolerating high biological protein from animal sources [2,6,24,25]. Although most of our patients achieved the EPG total protein recommendation, we suggest that the NP tolerance should be considered within the guidelines [6,26]. A higher NP intake sourced from high biological foods is likely to be associated with a more efficient utilization so there should be less need for a higher protein intake. Patients with milder forms of PKU who are less reliant on PE from PS should have similar protein requirements to the general population. Therefore, the dietary PS requirement is likely to differ between HPA, mild and classical PKU patients. In contrast, patients with classical PKU are very dependent on PE from PS to attain their TPA requirements.

In our study, PS was given, at least in three daily doses. Evidence suggests that an increased number of daily PS doses even helps mirror the slow protein digestion mechanism of NP and is associated with better protein utilization and improves Phe tolerance [25,27]. However, the higher the number of daily doses of PS intake, the more challenging it is for patients, leading to poor adherence and omitted PS doses [11,27,28]. In addition, low adherence with PS may adversely affect metabolic control and growth. We have a mixed cohort of patients with anthropometry and metabolic control data. Even though it was not our aim to describe growth and development, our sample had good growth with acceptable BMI, a reflection of a satisfactory protein intake and continuous nutritional vigilance. This result has also been demonstrated in other publications by this group, which describes the importance of frequent monitoring including biochemical markers and body composition [29,30].

Blood Phe is the primary biomarker for monitoring metabolic control. This marker is essential to guide nutritional treatment, avoid poor outcomes, and assess patient adherence [6]. In our study, we found that patient blood Phe control was influenced by their PKU phenotype and treatment methodology (±BH4), patients with mild PKU (±BH4) having better blood Phe control than classical PKU patients. Metabolic control did not appear influenced by the dose of PE from PS. However, this patient cohort had acceptable median metabolic control for all age groups, although only 30% of patients with classical PKU achieved median blood Phe level within target range defined by the Portuguese Consensus [6,20]. Some authors have described that patients with PKU, with acceptable blood Phe control, may tolerate more NP than they have been prescribed [31,32,33]. It may be possible to increase NP intake by following a systematic program to establish maximal individual Phe tolerance [34]. In PKU, it is essential to perform at least one annual review of daily protein intake in order to prevent over-restriction of NP intake, especially in the pediatric group [14,32,35].

Our findings are in line with Leuzzi et al., who suggested the need for personalized treatment aimed at minimizing burden of care and costs of overtreatment in PKU. Only a regular systematic nutritional review will provide the data needed to reassess the protein requirements of each patient and will allow an individualized prescription in the terms of growth, metabolic control and general health [36]. Furthermore, we focus on the importance of further guidelines specifying protein quality according to PKU phenotype/Phe tolerance and the relevance of critically reanalyzing protein intake to minimize the synthetic characteristics of the Phe-restricted diet.

This study has some limitations. Dietary intake assessments are generally inaccurate, irrespective of the method used. Our patient self-reported data were obtained through a 24 h recall/food intake history, and dietary assessments might not correspond to the real NP/PS intakes of patients. Self-reports of dietary treatment either tend to under or overestimate nutritional intake [28]. A more controlled study using 3-day dietary registry would improve data accuracy in order to provide more detailed information on food intake. Clear differences were noted on food patterns across different levels of PKU severity. In future research, it would be important to analyze the ratio of vegetable/animal sources from NP intake. Aspects such as protein digestibility, energy intake, and the protein:energy ratio and their impact on the food pattern and outcomes should be investigated in the PKU population.

## 5. Conclusions

This study showed that the majority of patients met the EPG recommendations for total protein requirements. When this was not achieved, it was related to suboptimal adherence with PS or patients with mild PKU, who tolerate more NP and had less need for PS. Our study underlines the different food patterns consumed by patients with PKU, and how it was influenced by the variable phenotypes and the use of BH4. Applying a “one-size-fits-all” solution might not be suitable for every patient. In clinical practice, nutritional management should be individualized with systematic nutritional monitoring to this heterogeneous population with very different nutritional needs and food patterns.

## Figures and Tables

**Table 1:** General descriptive subject data.

Sample Size	Age	Gender	Disease Classification
N = 99	19.3 ± 8.2 years	Male: n = 53	HPA PKU: n = 24
	<12 years: 7.2 ± 2.6 years	Female: n = 46	Mild PKU: n = 48
	≥12 years: 22 ± 6.3 years		Classical PKU: n = 27

Abbreviations: HPA—hyperphenylalaninemia; PKU—phenylketonuria.

**Table 2:** Anthropometry data—weight (kg), height (cm) and BMI (kg/m^2^)—according to PKU severity (<12 years and ≥12 years).

Anthropometry Data		HPA		Mild PKU		Classical PKU	
		<12 years (n = 6)	≥12 years (n = 18)	<12 years (n = 12)	≥12 years (n = 36)	<12 years	≥12 years (n = 27)
Weight (kg)	Mean ± SD	27.9 ± 11.1	60.6 ± 13.9	28.5 ± 11.5	62.9 ± 14.2	--	62.0 ± 13.4
	z-score (<10 y)	0.4 ± 0.64	--	0.49 ± 1.27	--	--	--
Height (cm)	Mean ± SD	126 ± 17.1	165.9 ± 7.78	127.8 ± 18.5	164.9 ± 11.0	--	163.3 ± 8.06
	z-score (<19 y)	0.28 ± 0.88	−0.01 ± 1.16	0.42 ± 0.95	−0.02 ± 1.18	--	−1.01 ± 0.29
BMI (kg/m^2^)	Mean ± SD	16.9 ± 2.41	21.9 ± 4.12	16.8 ± 2.17	22.9 ± 3.78	--	22.3 ± 6.05
	z-score (<19 y)	0.34 ± 0.8	−0.1 ± 1.41	0.33 ± 1.11	−0.18 ± 1.0	--	−0.18 ± 1.71

Abbreviations: HPA—hyperphenylalaninemia; PKU—phenylketonuria; BMI—body mass index; SD—standard deviation.

**Table 3:** Median [P25–P75] protein intake (TP, PS and NP) according to age, PKU severity and type of treatment.

		TP Intake (g/kg) Median [P25–P75]	PS Intake (g/kg) Median [P25–P75]	NP Intake (g/kg) Median [P25–P75]
Age	<12 years (n = 18)	1.68 [1.42–2.03]	0.9 [0.05–1.06]	0.6 [0.49–1.65]
	≥12 years (n = 81)	1.42 [1.17–1.71]	0.67 [0–0.87]	0.73 [0.39–1.4]
PKU severity	HPA (n = 24)	1.8 [1.42–2.08]	0 [0–0]	1.8 [1.42–2.08]
	Mild (n = 48)	1.53 [1.29–1.73]	0.78 [0.26–1.02]	0.68 [0.48–1.15]
	Classical (n = 27)	1.28 [1.12–1.41]	0.87 [0.72–0.98]	0.34 [0.25–0.49]
Type of treatment	Diet only (n = 82)	1.43 [1.24–1.79]	0.72 [0–0.98]	0.6 [0.35–1.45]
	Diet + BH4 therapy (n = 17)	1.57 [1.29–1.71]	0.5 [0.25–0.78]	1 [0.79–1.2]

Abbreviations: PKU—phenylketonuria; HPA—hyperphenylalaninemia; BH4—tetrahydrobiopterin; TP—total protein; PS—protein substitutes and NP—natural protein.

**Table 4:** Relative frequency (%) of patients with different intake of TPA according to PKU severity.

PKU Severity	TPA	Pediatrics (<12 Years) n = 18	Adolescents (12–18 Years) n = 32	Adults (>19 Years) (n = 49)	Total Percentage of Patients
HPA (N = 24)	TPA < 100%	1/24 (4%)	1/24 (4%)	1/24 (4%)	12%
	TPA ≥ 100–120%	-	1/24 (4%)	---	4%
	TPA ≥ 120–140%	-	---	---	
	TPA ≥ 140%	5/24 (21%)	11/24 (46%)	4/24 (17%)	84%
MILD PKU (N = 48)	TPA < 100%	--	---	1/48 (2%)	2%
	TPA ≥ 100–120%	--	---	3/48 (6%)	6%
	TPA ≥ 120–140%	---	2/48 (4%)	2/48 (4%)	8%
	TPA ≥ 140%	12/48 (25%)	11/48 (23%)	17/48 (36%)	74%
CLASSICAL PKU (N = 27)	TPA < 100%	--	---	2/27(7%)	7%
	TPA ≥ 100–120%	--	---	1/27 (4%)	4%
	TPA ≥ 120–140%	--	2/27(7%)	5/27 (19%)	26%
	TPA ≥ 140%	--	4/27 (15%)	13/27 (48%)	67%

Abbreviations: PKU—phenylketonuria; HPA—hyperphenylalaninemia and TPA—total protein adequacy.

**Table 5:** Median TPA (%), median PS contribution (%) to WHO recommendations (%) of patients according to patients age, PKU severity and type of treatment.

		TPA (%) Median [P25–P75]	PS Contribution (%) Median [P25–P75]	NP Contribution (%) Median [P25–P75]
Age	<12 years (n = 18)	184 [158–223]	96 [6–117]	73 [55–183]
	≥12 years (n = 81)	166 [140–201]	80 [0–105]	88 [17–164]
PKU severity	HPA (n = 24)	202 [165–230]	0 [0–0]	202 [165–234]
	Mild (n = 48)	178 [151–204]	91 [31–117]	78 [57–133]
	Classical (n = 27)	154 [135–170]	105 [86–117]	40 [30–59]
Type of treatment	Diet only (n = 82)	168 [144–202]	86 [0–116]	69 [41–169]
	Diet + BH4 (n = 17)	182 [155–206]	66 [30–90]	121 [89–143]

Abbreviations: PKU—phenylketonuria; HPA—hyperphenylalaninemia; BH4—tetrahydrobiopterin; TPA—total protein adequacy; PS—protein substitutes; NP—natural protein.

**Table 6:** Patients’ median [P25–P75] blood Phe metabolic control (µmol/L < 12 years and ≥12 years) and % of blood [Phe] within the Portuguese Consensus Recommendation according to age, PKU severity and type of treatment.

		Median µmol/L [P25–P75] <12 Years	Median µmol/L [P25–P75] ≥12 Years	Median % of Blood Phe Levels within Target Range
Age	<12 years (n = 18)	294 [234–354]	--	65 [50–83.0]
	≥12 years (n = 81)	--	450 [270–600]	61 [15.4–100]
PKU severity	HPA (n = 24)	247 [234–279]	217 [186–249]	100 [98.3–100]
	Mild (n = 48)	331 [257–360]	462 [313–553]	57 [22.7–92.8]
	Classical (n = 27)	--	610 [462–806]	17 [0–55.9]
Type of treatment	Diet only (n = 82)	295 [236–344]	462 [240–636]	59 [15.5–100]
	Diet + BH4 (n = 17)	296 [266–325]	421 [347–503]	77 [50.9–94.1]

Abbreviations: PKU—phenylketonuria; HPA—hyperphenylalaninemia; BH4—tetrahydrobiopterin; Phe—phenylalanine.

## Data Availability

The data will be made available from the authors upon reasonable request.
